# Analgesic efficacy of bilateral erector spinae plane block in pediatric tethered cord syndrome surgery: a prospective, randomized controlled trial

**DOI:** 10.1590/1806-9282.20260443

**Published:** 2026-08-10

**Authors:** Sermin Eminoglu, Elif Basaran Gundogdu, Seyda Efsun Ozgunay, Asiye Demirel, Nermin Kilicarslan, Serpil Ekin, Anıl Onur, Mesut Öztürk

**Affiliations:** 1 University of Health Sciences, Bursa Yuksek Ihtisas Education and Research Hospital, Department of Anesthesiology and Reanimation – Bursa, Turkey.; 2 University of Health Sciences, Bursa Yuksek Ihtisas Education and Research Hospital, Department of Brain and Nerve Surgery – Bursa, Turkey.

**Keywords:** Children, Pain management, Tethered cord syndrome, Nerve block, Analgesia, Postoperative

## Abstract

**OBJECTIVE:**

The aim of this study was to evaluate the analgesic efficacy of the ultrasound-guided erector spinae plane block in pediatric patients undergoing surgery for tethered cord syndrome.

**METHODS:**

In a randomized, prospective, single-blind study, 73 patients aged 1–18 years with American Society of Anesthesiologists I–III status scheduled for tethered cord syndrome surgery were divided into control and erector spinae plane block groups. In the erector spinae plane block group, 0.5 mL kg^-1^ of 0.25% bupivacaine was administered under ultrasound guidance. Pain assessment scores were recorded in the recovery unit during the first 20 min and on the ward during the first 24 h. The groups’ need for rescue analgesia, intraoperative anesthetic drug consumption, and the time to the first postoperative analgesic request were investigated.

**RESULTS:**

The erector spinae plane block group had lower pain scores (up to approximately 4 h postoperatively), anesthetic and opioid consumption, and rescue analgesia requirements. A significant difference was observed in the time to first analgesic requirement. In total, 80.6% of patients in the control group still required additional analgesia at 30 min.

**CONCLUSION:**

The study demonstrated that erector spinae plane block delayed the time to first analgesic requirement and provided adequate postoperative analgesic effect in pediatric tethered cord syndrome surgery.

## INTRODUCTION

Tethered cord syndrome (TCS) is a developmental anomaly of the neuroaxis characterized by tension on the spinal cord due to congenital or acquired adhesions^1^. Surgical release of the tight and tethered filum terminale is the most important treatment^2^. Intraoperative neurophysiological monitoring (IONM) is required for safe surgery^3^. Total intravenous anesthesia [TIVA-target-controlled infusion (TCI)] with propofol and remifentanil is used as the anesthetic method during the procedure.

Postoperative pain is acute pain caused by surgical trauma and signal transmission in afferent neurons. Postoperative pain management remains a significant problem in children. Ultrasound-guided erector spinae plane block (ESPB) is a new regional anesthesia technique developed by Forero et al. by injecting local anesthetic into the fascial plane between the erector spinae muscle and the transverse process to block spinal nerve branches^4^. In recent years, it has been reported to provide effective postoperative analgesia in various pediatric surgical patients^5–7^.

In this study, the primary outcome was the effect of bilateral ESPB on pain scores during the first 24 h postoperatively in pediatric patients undergoing surgery for TCS. Secondary outcomes included intraoperative anesthetic drug consumption, the need for rescue analgesia, the time to the first postoperative analgesic request, as well as hemodynamic and clinical data.

## METHODS

This prospective, randomized controlled, single-blind clinical study was approved by the local ethics committee with protocol number Decision No: 2025-7/12 (April 8, 2025). It was conducted at Bursa Training and Research Hospital between April 8, 2025, and December 1, 2025. It was registered on Clinicaltrials.gov under the number NCT07404995. The principles outlined in the Helsinki Declaration and the CONSORT standards were followed. The parents/legal guardians of the patients, as well as children aged 3–8 and 9–18, who were capable of giving consent, provided separate written consent for themselves and their parents/legal guardians. Pediatric patients aged 1–18 who were scheduled to undergo surgery due to TCS and had an ASA physical status of I–III were included. Patients who did not provide consent, had ASA IV–V status, were taking anticoagulant medication, had an allergy to local anesthetic drugs, or had an infection in the area where the block was planned were excluded from the study.

Patients were randomly assigned to groups (control and ESPB) by computer in a 1:1 ratio. Sealed envelopes were used. Perioperative monitoring and ESPB administration were performed by the same anesthesiologists and could not be blinded. For both groups, recovery room and ward nurses were trained on the FLACC (face, legs, activity, cry, and consolability) for patients under 5 years of age and the Wong-Baker FACES Pain Assessment Scale for patients 5 years of age and older (both scores are rated on a scale of 0 to 10, where 0 means no pain and 10 means the most severe pain). Notably, one anesthesiologist and these individuals were not informed about the groups and were blinded. The same surgical dressing was applied to both groups.

In anesthesia management, patients received midazolam premedication. Intraoperative vital signs were monitored with routine monitoring. Anesthesia induction was achieved with propofol, fentanyl, and rocuronium. All patients had a transcranial motor-evoked potentials (TcMEP) device attached by neurosurgeons in the IONM for TcMEP. During maintenance of anesthesia with TCI, propofol (2.5–4 μg mL^-1^) and remifentanil (0.5–1 μg kg^-1^) were adjusted, and infusion was initiated. No inhalation anesthetic was used, and no additional doses of muscle relaxant were administered. Perioperative vital signs and demographic data, along with the total intraoperative doses of propofol and remifentanil administered, were recorded by the researchers.

In the recovery room, the groups were assessed for pain scores every 5 min for 20 min, and patients with a score >5 received 0.5 μg kg^-1^ of fentanyl administered by the researchers for rescue analgesia, and the results were recorded.

In the ward, the patients’ pain scores in the first 24 h postoperatively, the time of the first analgesic requirement, nausea-vomiting, and length of hospital stay were queried and recorded. For postoperative analgesia management and as needed, paracetamol 15 mg kg^-1^/6 h was administered intravenously.

### Erector spinae plane block procedure

Under ultrasound guidance (USG—Xperius, Braun-Philips) in the prone position, a linear probe was used with a depth of 2–4 cm and a frequency of 10–15 mHz. Prior to the surgical incision, the probe was positioned approximately 2–3 cm lateral to the spinous process on a segment of the surgical site (L3 or L4) in the parasagittal plane. After visualizing the transverse process using an in-plane approach, a 50-mm-long block needle (Braun stimuplex® Braun, Germany) was inserted through the skin. After passing through the trapezius and erector spinae muscles, when the needle reached the transverse process (at a depth of approximately 2–3 cm), a test dose of 0.5–1 mL of 0.9% NaCl was administered between the erector spinae muscle fascia and the vertebral transverse process to confirm the needle location. After the fascia was opened, 0.5 mL/kg of 0.5% bupivacaine local anesthetic was diluted 1:1 with 0.9% NaCl solution to prepare a 0.25% bupivacaine concentration; care was taken not to exceed the maximum safe dose limit of 2.5 mg/kg, and the volume was individualized according to the dose limit. The calculated solution was administered to the erector spinae area, and ESPB was performed. The same procedures were performed on the contralateral side. No complications were observed during or after the procedure. The control group received only intravenous analgesia without any regional block. In total, 15 mg kg^-1^ of paracetamol was administered intravenously to both groups 20 min before the end of surgery.

### Statistical analysis

The primary endpoint of the study was the postoperative pain score at 24 h after surgery. Pain was assessed using age-appropriate pain scales and transformed to a common 0–100 metric. Sample size calculation was performed using G*Power version 3.1.9.2. The expected effect size was based on a previously published randomized controlled trial evaluating bilateral and bilevel erector spinae plane block in pediatric idiopathic scoliosis surgery (Domagalska). In that study, postoperative NRS pain scores were assessed as the primary outcome. The reported 24-h NRS pain scores correspond to an estimated Cohen's d of approximately 1.96. To avoid overestimating the expected treatment effect, we used a more conservative effect size (Cohen's d=0.70) in the sample size calculation. With a two-sided alpha level of 0.05, 80% power, and an allocation ratio of 1:1, the required sample size was 34 patients per group, yielding a total of 68 patients. The Shapiro-Wilk test was used to assess the normality of continuous variables. Descriptive statistics are presented as medians (minimum–maximum) for quantitative variables and as frequencies and percentages for categorical variables.

For independent two-group comparisons of non-normally distributed continuous variables, the Mann-Whitney U test was used. Categorical variables were analyzed using Pearson's chi-square test, Fisher's exact test, or Fisher–Freeman-Halton test, as appropriate.

Repeated postoperative pain scores were analyzed using generalized estimating equations to account for within-subject correlations over time. The GEE model included group, time, and group×time interaction as model factors. A normal distribution with an identity link function, an exchangeable working correlation structure, and robust standard errors was used. Pairwise between-group comparisons at individual time points were performed using estimated marginal means and adjusted for multiple testing across the 12 predefined postoperative time points using the Bonferroni correction.

A p-value of <0.05 was considered statistically significant. Statistical analyses were performed using IBM SPSS Statistics for Windows, Version 29.0.2.0 (IBM Corp., Armonk, NY, USA).

## RESULTS

**Figure 1:**
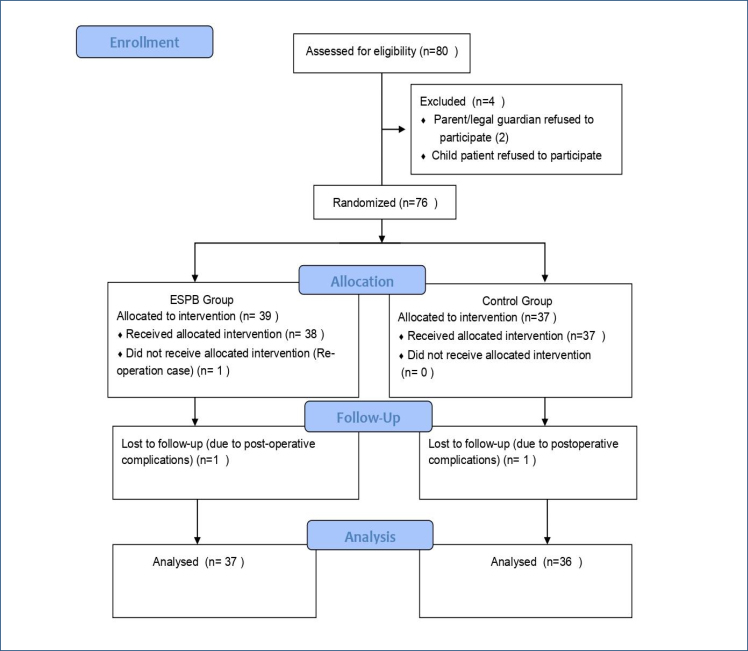
The CONSORT flow chart

A total of 80 pediatric patients were evaluated. Notably, seven patients were excluded from the study for various reasons. Data from the remaining 73 patients were analyzed (Figure 1).

No statistically significant differences were found between the groups in demographic and perioperative characteristics of the patients, as summarized in Table 1 (p>0.05 for all).

**Table 1:** Demographic and perioperative characteristics, postoperative pain scores over time, intraoperative analgesic/anesthetic consumption, and postoperative outcomes.

Variables		Control group (n=36)	ESPB group (n=37)	p-value
Age (years)		4 (1–16)	4 (1–16)	0.365
Sex (female)		24 (66.7%)	18 (48.6%)	0.119
ASA (I)		16 (44.4%)	17 (45.9%)	0.897
BMI		16.6 (6.9–41.7)	14.8 (10.0–25.5)	0.562
Anesthesia duration (min)		135 (65–225)	150 (85–300)	0.074
Surgery duration (min)		92.50 (30–210)	100 (30–240)	0.262
Crystalloid (mL)		250 (60–1,000)	180 (60–1,200)	0.934
Bleeding (mL)		10 (0–200)	10 (0–300)	0.905
Systemic disease, yes		22 (61.1%)	21 (56.8%)	0.705
Allergy, yes		1 (2.8%)	2 (5.4%)	1.000
Pain score				
	Recovery room 0 min	6 (0–10)	0 (0–6)	<0.001
	5 min	6 (0–10)	2 (0–8)	<0.001
	10 min	6 (1–10)	2 (0–10)	<0.001
	15 min	5 (2–10)	2 (0–10)	<0.001
	20 min	6 (2–10)	2 (0–8)	<0.001
	Ward 30 min	6 (2–10)	4 (0–10)	<0.001
	1 h	5 (2–8)	4 (0–8)	0.003
	2 h	4 (2–10)	3 (0–8)	0.033
	4 h	3.5 (0–9)	2 (0–6)	0.751
	6 h	2 (0–8)	2 (0–10)	1.000
	12 h	1 (0–10)	2 (0–6)	1.000
	24 h	0 (0–6)	0 (0–6)	1.000
	Total propofol consumption (mg)	506.5 (155–1,700)	382 (168–1,282)	0.039
	Total remifentanil consumption (mcg)	465 (110–1,600)	240 (50–1,200)	<0.001
	Hospital stay, days	2 (1–4)	2 (2–3)	0.188
	Nausea, yes	3 (8.3%)	3 (8.1%)	1.000
	Vomiting, yes	2 (5.6%)	2 (5.4%)	1.000
	Rescue analgesia, yes	12 (33.3%)	2 (5.4%)	0.002
	Medication (fentanyl), n/N	12/12 (100%)	2/2 (100%)	-
Post-stage				
	30 min	29 (80.6%)^a^	9 (24.3%)^b^	<0.001
	1 h	5 (13.9%)	8 (21.6%)	
	2 h	2 (5.6%)	7 (18.9%)	
	4 h	0 (0.0%)^a^	5 (13.5%)^b^	
	6 h	0 (0.0%)	2 (5.4%)	
	12 h	0 (0.0%)^a^	5 (13.5%)^b^	
	24 h	0 (0.0%)	1 (2.7%)	
Medication used				
	Paracetamol	36 (100.0)	36 (97.3)	1.000

Data for demographic and perioperative characteristics are presented as descriptive statistics: median (minimum–maximum), frequency (n), and percentage (%). Mann-Whitney U, Pearson chi-square, and Fisher's exact chi-square tests were used to compare the data. Data on the change in postoperative pain over time are presented as median (minimum–maximum). Repeated postoperative pain scores were analyzed using generalized estimation equations with group, time, and group×time interaction as model factors. A modifiable study correlation structure with robust standard errors was used. Pairwise within-group comparisons at each time point were adjusted for multiple testing using Bonferroni correction across 12 postoperative time points. Descriptive statistics for intraoperative analgesic/anesthetic consumption and postoperative outcomes are presented as medians (minimum–maximum), frequency (n), and percentage (%). Mann-Whitney U, Pearson chi-square, Fisher's exact chi-square, and Fisher-Freeman-Halton tests were used to compare data. The Bonferroni test was used for pairwise comparisons. Superscripts "a" and "b" indicate the results of pairwise comparisons between groups, where different superscript letters indicate a significant difference between groups. ESPB: erector spinae plane block; ASA: American Society of Anesthesiologists; BMI: body mass index.

Postoperative pain scores were analyzed using generalized estimating equations with a flexible correlation structure and robust standard errors (Table 1). The model included group, time, and group×time interaction. The analysis revealed a significant group effect (Wald χ²=29.801, df=1, p<0.001), a significant time effect (Wald χ²=259.452, df=11, p<0.001), and a significant group×time interaction (Wald χ²=72.774, df=11, p<0.001). These findings indicate that postoperative pain scores changed differently over time between the ESPB and control groups.

**Figure 2:**
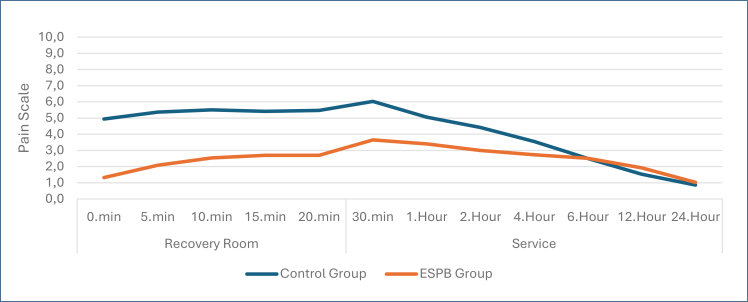
The comparison of pain scores

In post-hoc pairwise comparisons, pain scores were significantly lower in the ESPB group from admission to the recovery room until the 2nd postoperative hour. Upon admission to the recovery unit (0 min), the median pain score was 0 (0–6) in the ESPB group, whereas it was 6 (0–10) in the control group. From the 4th postoperative hour onward, no statistically significant difference was observed between the groups (p=0.751) (Figure 2). The total intraoperative consumption of propofol and remifentanil was significantly lower in the ESPB group (p=0.039, p<0.001, respectively) (Table 1).

No statistically significant difference was found between the groups in terms of intraoperative vital signs at any time point (p>0.05).

Postoperative clinical data and additional analgesic requirements are summarized in Table 1. There was no statistical difference between the groups in hospital stay, nausea, or vomiting rates (p>0.05). However, the rate of rescue analgesia requirement in the ESPB group was significantly lower (5.4 vs. 33.3%) (p=0.002). Only fentanyl was used as rescue analgesia in both groups. There was a highly significant difference between the groups in the time to first analgesic requirement (p<0.001). While the vast majority of patients in the control group (80.6%) required additional analgesia within 30 min, this need was delayed to much later hours in the ESPB group.

## DISCUSSION

In this randomized controlled trial, the ESPB block in pediatric patients undergoing surgery for TCS reduced pain ratings in the recovery unit and up to the 4th postoperative hour, reduced intraoperative anesthetic drug consumption and the need for rescue analgesia, and prolonged the time to the first postoperative analgesic requirement. It did not affect hospital stay, nausea, or vomiting.

Treatment for tethered cord involves surgical detethering, which includes extensive muscle dissection along with a wide laminectomy. Following Forero's description, applications for postoperative pain in the pediatric population began in 2017^8^. Very few randomized controlled trials have reported ESPB outcomes in pediatric patients^5–7,9^. Mogahed et al. applied bilateral ESPB in pediatric cardiac surgery. They reported that postoperative pain scores, including at 10 h, were higher in the control group^9^. Mostafa et al. also applied ESPB in pediatric patients undergoing spleen surgery. They found that the postoperative pain score was lower in the first 8 h^10^. ESPB has recently been used in spinal surgery for postoperative analgesia^11^. Diwan et al. applied ESPB in pediatric scoliosis surgery. In this series of six disease cases, they reported that a single injection of ESPB provided effective perioperative analgesia^12^. Domagalska et al. compared bilateral and bilevel ESPB with a placebo block in thoracic scoliosis surgery in patients aged 10–18 years and found that the ESPB group had lower pain scores at all time points during the 48-h postoperative period^8^. Kamel et al. applied ESPB in patients aged 12–25 years undergoing scoliosis repair and reported better pain control^13^. In their meta-analysis, Luo et al. stated that the pain score was lower in the ESPB group 0 and 6 h after surgery, with no difference between the two groups at 12 and 24 h^14^. In our study, the youngest patient who underwent surgery was 1 year old, and the oldest was 18 years old. When patients were evaluated using age-appropriate postoperative pain assessment scales, we found that upon admission to the recovery room (0 min), the median pain score was 0 (0–6) in the ESPB group versus 6 (0–10) in the control group, and pain ratings remained low in the ward until the 4th postoperative hour. We can state that our results are similar to those reported in the meta-analyses conducted by Luo and Singh^11,12^. In those studies, the difference between groups was resolved on average by 6 h postoperatively, whereas in our study, this occurred by 4 h. We believe that the reason for this difference in duration compared to other studies may be the routine administration of 15 mg/kg intravenous paracetamol every 6 h postoperatively to both groups as part of the analgesic strategy.

Most studies evaluated rescue analgesia and the time to request initial analgesia. Notably, one study found that rescue analgesia was required in approximately 315 min in the ESPB group compared to approximately 37 min in the control group, and that the total number of rescue analgesics was higher in the control group^7^. Singh et al. also reported that the duration of FLACC score ≥4 was 360±30 min compared to 160±25 min^12^. Another study reported that the rescue analgesic request time was 70/16 min on average in the ESPB/control group^11^. Notably, two studies that conducted systematic reviews and meta-analyses concluded that ESPB prolonged the time to the first request for rescue analgesia and reduced the number of patients requiring rescue analgesia. Children receiving ESPB were approximately 10 times more likely to avoid the need for rescue opioids^11,13^. Our study yielded results consistent with those in the literature. The rate of rescue analgesia requirement was significantly lower in the ESPB group (5.4%) compared to the control group (33.3%). A highly significant difference was found between the groups in terms of the first analgesic requirement, and the vast majority of patients in the control group (80.6%) required additional analgesia by the 30th minute.

Among the secondary outcomes of our study, we investigated whether ESPB reduces intraoperative anesthetic drug consumption. During this period, when neuromuscular blockers are not used, the doses of anesthetic drugs are particularly important in pediatric patients. Propofol infusion requires particular attention in low-weight children. Similarly, remifentanil infusion is important in terms of intraoperative hemodynamic management and perioperative opioid side effects. The results of surgeries with neuromonitoring are particularly important for our study. Domagalska et al. reported that ESPB reduced total opioid consumption and the need for intraoperative remifentanil and propofol in pediatric scoliosis surgery^6^. Kamel et al. also found that ESPB significantly reduced total intraoperative fentanyl consumption in patients undergoing scoliosis repair compared to the control group^10^. In our study, similar results were observed, with total intraoperative propofol and remifentanil consumption being significantly lower in the ESPB group compared to the control group. Due to the physiological differences in pediatric patients, it has been noted that monitoring pain during the intraoperative period based solely on hemodynamic changes is more challenging than in adult patients. A study investigated the necessity of pain monitors in a group of pediatric patients scheduled for abdominal surgery and undergoing ESPB^14^. Although hemodynamic data and pain monitor data were consistent with each other following a pain alert, it was noted that pain monitor data were less influenced by other variables and provided the clinician with clearer information regarding pain. In our study, a pain monitor was not used; the administration of propofol and remifentanil was successfully managed in both groups through BIS monitoring, hemodynamic changes, and feedback from IONM providers.

Tethered cord surgery is considered a safe procedure with a relatively short postoperative hospital stay and low complication rate^2^. In our study, the necessary follow-up until discharge after TCS surgery was routinely planned for 3 days in all patients by the surgeon. Additionally, postoperative analgesia was routinely administered with paracetamol every 6 h. These circumstances explain the absence of a statistically significant difference between the groups in our study in terms of hospital stay, nausea, and vomiting.

Our study has some limitations. In this randomized study, the researchers, block practitioners, and intraoperative monitors could not be blinded. Although our sample size had sufficient power for the primary outcome, the study was conducted with a small sample at a single center. Another limitation is that in this surgery, performed under IONM, pain monitoring is required in addition to BIS in anesthesia monitoring, which we did not use.

## Data Availability

The datasets generated and/or analyzed during the current study are available from the corresponding author upon reasonable request.
